# Reply to Weiermayer et al. Evidence-Based Human Homeopathy and Veterinary Homeopathy. Comment on “Bergh et al. A Systematic Review of Complementary and Alternative Veterinary Medicine: “Miscellaneous Therapies”. *Animals* 2021, *11*, 3356”

**DOI:** 10.3390/ani12162098

**Published:** 2022-08-17

**Authors:** Anna Bergh, Iréne Lund, Anna Boström, Heli Hyytiäinen, Kjell Asplund

**Affiliations:** 1 Department of Clinical Sciences, Swedish University of Agricultural Sciences, SE 750 07 Uppsala, Sweden; 2 Department of Physiology and Pharmacolgy, Karolinska Institutet, SE 171 77 Stockholm, Sweden; 3 Department of Equine and Small Animal Medicine, Faculty of Veterinary Medicine, University of Helsinki, P.O. Box 57, 00014 Helsinki, Finland; 4 Department of Public Health and Clinical Medicine, Umeå University, SE 901 87 Umeå, Sweden

We appreciate the interest in our article *A Systematic Review of Complementary and Alternative Veterinary Medicine: “Miscellaneous Therapies”* published in *Animals,* Volume 11 [1]. Our article is one in a comprehensive series of systematic reviews of original research on complementary and alternative veterinary medicine (CAVM) methods used in sport and companion animals. The commentators refer to research on homeopathy in humans and farm animals [2]. The scope of our systematic literature review was to study the scientific documentation for equine, canine and feline species. As is evident from our systemic review, the scientific documentation, irrespective of clinical condition, provides no reliable evidence for the clinical efficacy of homeopathy in the treatment of dogs, cats and horses.
